# Telomere-Binding Protein TPP1 Modulates Telomere Homeostasis and Confers Radioresistance to Human Colorectal Cancer Cells

**DOI:** 10.1371/journal.pone.0081034

**Published:** 2013-11-19

**Authors:** Lei Yang, Wenbo Wang, Liu Hu, Xiaoxi Yang, Juan Zhong, Zheng Li, Hui Yang, Han Lei, Haijun Yu, ZhengKai Liao, Fuxiang Zhou, Conghua Xie, Yunfeng Zhou

**Affiliations:** 1 Hubei Cancer Clinical Study Center, Hubei Key Laboratory of Tumor Biological Behaviors, Zhongnan Hospital, Wuhan University, Wuhan, China; 2 Department of Radiation Oncology & Medical Oncology, Zhongnan Hospital, Wuhan University, Wuhan, China; 3 Department of Oncology, the Fifth Hospital of Wuhan, Wuhan, China; Texas Tech University Health Sciences Center, United States of America

## Abstract

**Background:**

Radiotherapy is one of the major therapeutic strategies in cancer treatment. The telomere-binding protein TPP1 is an important component of the shelterin complex at mammalian telomeres. Our previous reports showed that TPP1 expression was elevated in radioresistant cells, but the exact effects and mechanisms of TPP1 on radiosensitivity is unclear.

**Principal Findings:**

In this study, we found that elevated TPP1 expression significantly correlated with radioresistance and longer telomere length in human colorectal cancer cell lines. Moreover, TPP1 overexpression showed lengthened telomere length and a significant decrease of radiosensitivity to X-rays. TPP1 mediated radioresistance was correlated with a decreased apoptosis rate after IR exposure. Furthermore, TPP1 overexpression showed prolonged G2/M arrest mediated by ATM/ATR-Chk1 signal pathway after IR exposure. Moreover, TPP1 overexpression accelerated the repair kinetics of total DNA damage and telomere dysfunction induced by ionizing radiation.

**Conclusions:**

We demonstrated that elevated expressions of TPP1 in human colorectal cancer cells could protect telomere from DNA damage and confer radioresistance. These results suggested that TPP1 may be a potential target in the radiotherapy of colorectal cancer.

## Introduction

Colorectal cancer (CRC), with over 1.2 million new cases and 608,700 deaths in 2008, is a major cause of cancer-related death in many countries [1]. Radiotherapy is one of the major therapeutic strategies in colorectal cancer treatment with effective local control, protection of normal tissues and less systemic effects [2,3]. However, many patients still experience recurrence or metastasis after radiation treatment. The main cause of radiotherapy failure is cellular radioresistance. So identifying new factors that predict radioresistance is an area of intense research and could be of great value in the treatment of cancers. 

Telomeres are specialized DNA-protein complexes at the ends of eukaryotic chromosomes composed of a variable number of tandemly repeated TTAGGG sequences and associated proteins [4]. Telomeres play crucial roles in ensuring genomic stability and integrity [5-8]. Moreover, studies have clarified that telomere homeostasis serves as a potential target in cancer treatment, especially in radiotherapy [9-12]. Our previous research also indicated that there was a significant negative correlation of telomere length and radiosensitivity and telomere length may be used as a promising tool to predict the radiosensitivity of human carcinomas [13]. 

Telomere homeostasis is affected by multiple elements, and one of the major regulators is shelterin. The shelterin complex consists of six telomere-associated proteins: TRF1, TRF2, RAP1, TIN2, TPP1 and POT1 [8]. Disruption in the shelterin would lead to telomere dysfunction and, potentially, chromosomal instability [14]. TPP1 (also known as TINT1/PTOP/PIP1) is a critical member of shelterin and associates with other telomere-binding proteins to form the core shelterin [8]. TPP1 heterodimerizes with POT1 and enhances its affinity with telomere ssDNA [15,16]. The POT1-TPP1 complex is capable of recruiting and stimulating telomerase activity, thereby regulating telomere length through TPP1-telomerase interaction [17-19]. Previous researches demonstrated that TPP1 knockdown activates an ATM-dependent DNA damage response marked by the formation of telomere dysfunction-induced foci (TIFs) at telomeres [20]. Moreover, we observed that TPP1 expression was elevated in radioresistant cells and TPP1 may involve in cancer radioresistance [21]. However, the exact effects and mechanism of TPP1 on radiosensitivity is unclear.

To further clarify the functions of TPP1, we investigated the role of TPP1 overexpression on radiosensitivity and telomere homeostasis in human colorectal cancer cells in this study. 

## Materials and Methods

### Cell Culture and Treatment

Human colorectal cancer cell lines (HCT116, SW480, LoVo, HT29 and DLD-1) were purchased from the Cell Bank of the Chinese Academy of Science, Shanghai, China. All cells used in this study were cultured in 1640 medium supplemented with 10% fetal bovine serum. HCT116 cells were transfected with pcDNA6-flag-hTPP1 (a kind gift from Dr Joachim Lingner) [19] or pcDNA6 empty vector (Invitrogen) using lipofectamine 2000 (Invitrogen). TPP1 overexpressing cells were selected with 5 ug/ml blasticidin (sigma) for 4 weeks. The stable transfection cell lines were named as HCT116-TPP1 and HCT116-Mock, respectively. 

X-rays irradiation was performed with a X-rays generator (Primus High-Energy Siemens), emitting at a fixed dose rate of 2 Gy/min, energy of the X-rays used to irradiate cells is 0-10 Gy.

### Clonogenic Assay

The cells were plated in 6-well plate culture flasks. After 24 h, cells were irradiated with graded doses (0, 2, 4, 6, 8, 10 Gy) using X-ray generator (Primus High-Energy Siemens) at a dose rate of 2 Gy/min. The cells were then cultured in an incubator containing 5% CO2 at 37 °C for 14 days. The subsequent steps and calculation of the surviving fraction were performed as previously described [13].All experiments were done at least thrice.

### Flow Cytometry Analysis of Cell Cycle

Briefly, cells were exposed to 6 Gy IR and then incubated for the indicated times (0, 6, 12, 18, 24, 30, 36, and 42 h), then cell cycle analyses were performed as previously described [21]. DNA histograms were analysed using Modifit software. Experiments were performed in triplicate.

### Flow Cytometry Analysis of Apoptosis

Apoptosis assay was performed using an annexinV-FITC apoptosis detection kit (Beyotime, China) according to the manufacturer's instruction. Fluorescence was measured using a flow cytometer (Beckman Coulter, Brea, CA) and the data were analyzed with Cell Quest software. All samples were assayed in triplicate.

### Antibodies and Western Blot Analysis

Western blot was performed as previously reported [21]. Following antibodies are used in this study: TPP1 (Abcam), ATR, phospho-Ser345-Chk1/Chk1 and ATM (Cell Signaling Technology). A β-actin antibody (Santa cruz) was used to normalize loading differences between the samples. 

### Telomerase Activity Assay

The measurement of telomerase activity was carried out using the TRAP PCR ELISA kit (Roche) according to the manufacturer's instructions. The detailed method was performed as previously described [22].Sample absorbance was measured with a microplate reader (Bio-Rad) at the wavelength of 450/690 nm.

### Measurement of Telomere Length by Southern Blotting

Terminal restriction fragment determination was performed using the TeloTAGGG Telomere Length Assay kit (Roche) according to the manufacture’s instruction. Average telomere length (mean terminal restriction fragments length, TRF) was determined using the image analysis software (Bio-Rad). 

### Immunofluorescence

After indicated treatment, cells were fixed with 4% formaldehyde for 15 min and permeabilized with 0.2% Triton X-100 in PBS for 10 minutes at room temperature. After blocked with blocking solution, cells were incubated with the primary antibody overnight at 4°C then washed and incubated with the secondary antibody. Nuclei were stained with DAPI (Sigma) for 5 min at room temperature. Fluorescence signals were taken using a confocal microscope (Carl Zeiss LSM710).

### Telomere ChIP Assay and Dot Blot Analysis

Telomere ChIP Assay and Dot Blot Analysis were performed as previously reported [19]. After precipitation with the TRF2 antibody, DNA was purified from immunoprecipitated chromatin and blotted onto a Hybond-N membrane (Amersham), and telomere repeat sequences were detected with a TeloTAGGG telomere length assay kit (Roche Diagnostics). A nonspecific probe (Alu) was used as control. The signals were measured using image analysis software (Bio-Rad).

### Statistical Analysis

All data are expressed as mean ± SEM. Student's t-test was used to test statistical significance. P < 0.05 was considered to be significant. GraphPad Prism 5 (GraphPad Software, California) software was used as a statistical analysis tool.

## Results

### Correlation Between TPP1 Protein Expression, Telomere length and Intrinsic Radiosensitivity

**Figure 1:**
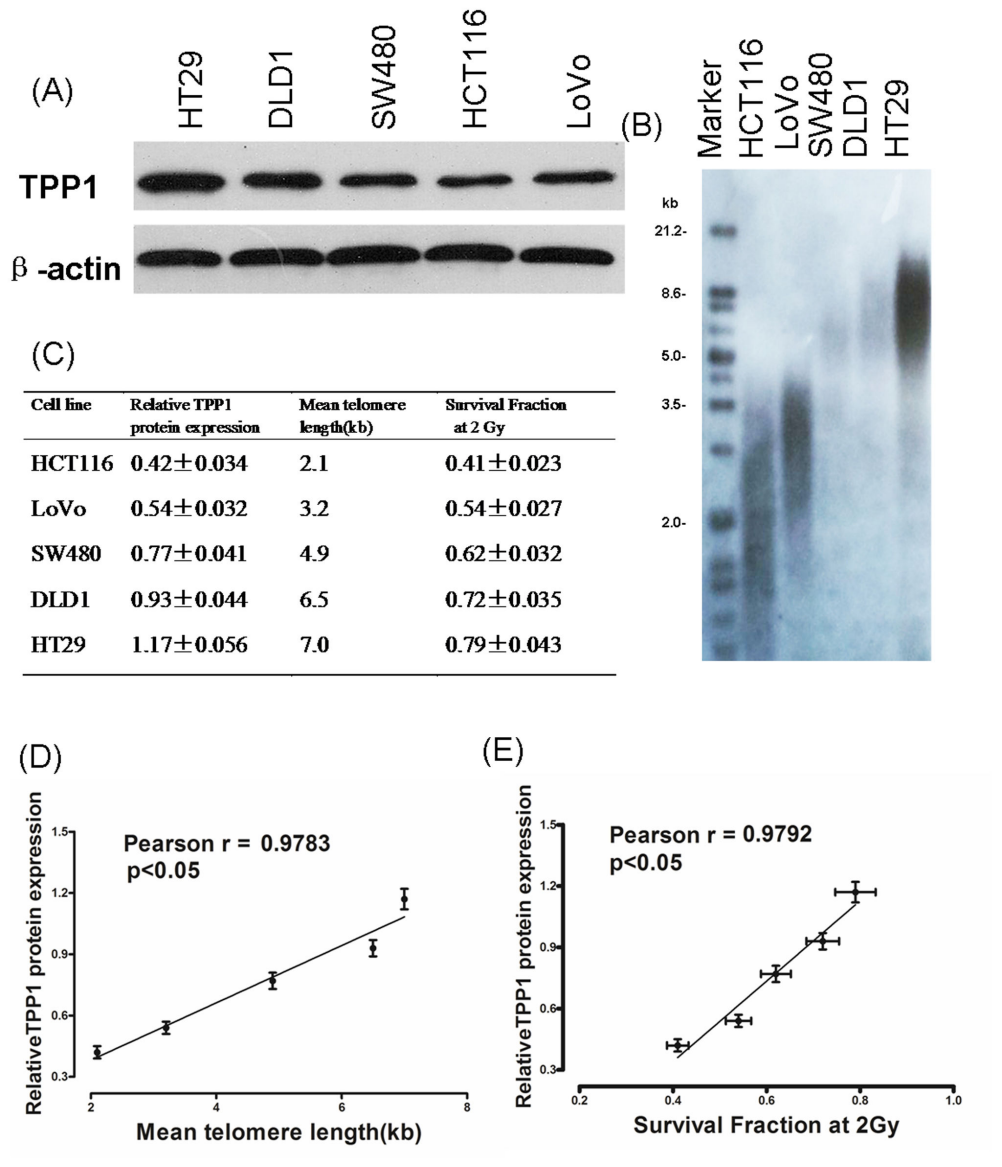
TPP1 production, radiosensitivity (SF2) and telomere length (TRF) in human colorectal cancer cell lines. (A) TPP1 production was detected by western blotting.. (B) Telomere length was examined by Southern blot analysis. (C) Relative TPP1 production, radiosensitivity (SF2) and telomere length (TRF) in human colorectal cancer cell lines. (D) Correlation between TPP1 production and radiosensitivity (SF2) in colorectal cancer cells was examined. (E) Correlation between TPP1 production and the TRF length in colorectal cancer cells was examined.

Firstly we examined TPP1 protein expression and telomere lengths in five colorectal cancer cells (Figure 1A and B). As shown in Figure 1D, TPP1 protein expression was significantly correlated with telomere length (R=0.9783, P < 0.05). Then cell survival was measured by a clonogenic assay and SF2 was used as an index of intrinsic radiosensitivity (Figure 1C). TPP1 expression was negatively correlated with intrinsic radiosensitivity(R = 0.9792, P < 0.05, Figure 1E). In summary, radioresistant cells have longer telomeres and higher production of TPP1 than that in radiosensitive cells. These result indicated that there was a significant correlation between TPP1 expression, telomere length and cell intrinsic radiosensitivity.

### TPP1 Overexpression Decreases Sensitivity of HCT116 Cells to Radiation

**Figure 2:**
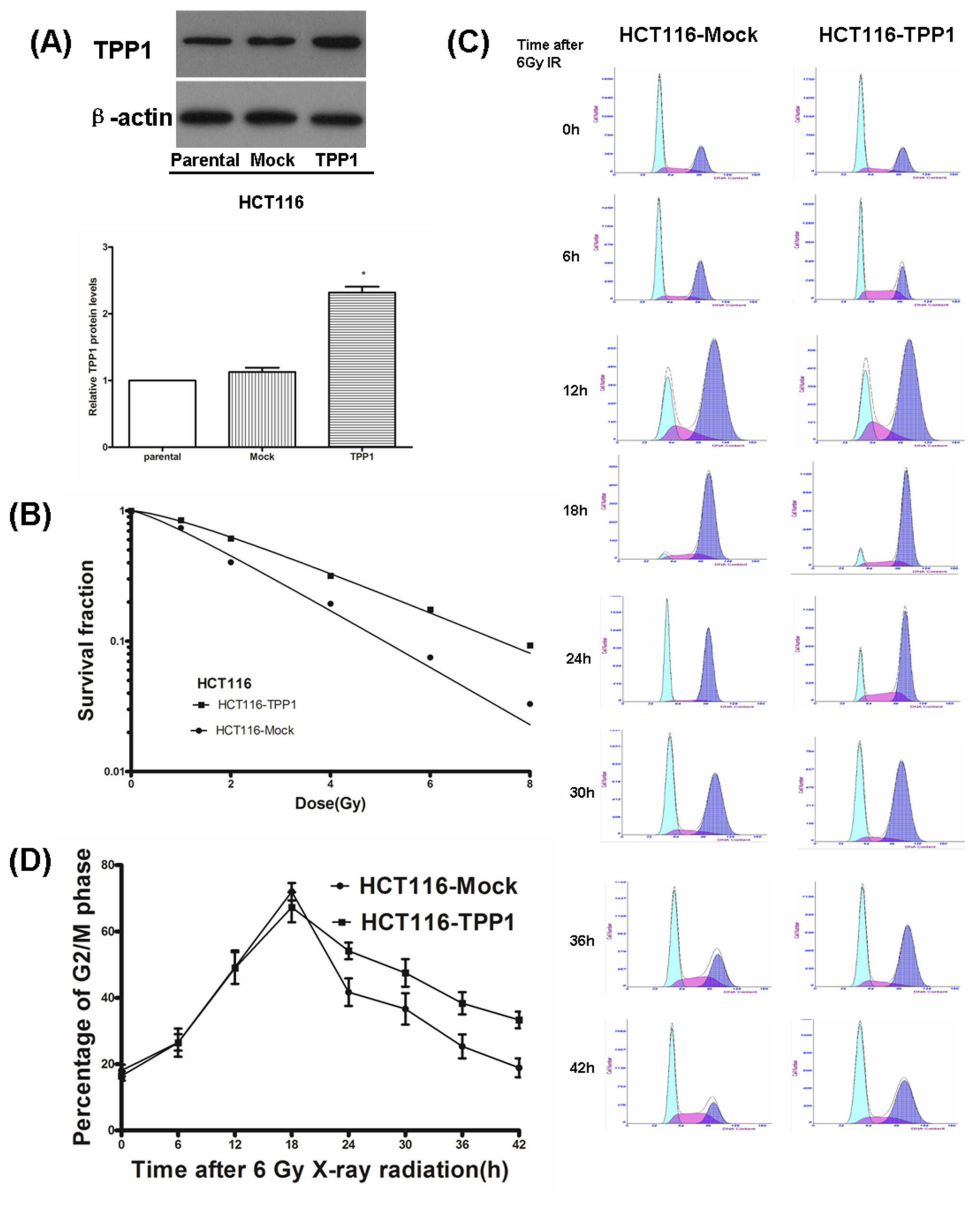
Effects of TPP1 overexpression on the radiosensitivity and cell cycle in HCT116 cells. (A)Verification of TPP1 overexpression by western blotting. (B) HCT116-Mock and-TPP1 cells were irradiated with X-rays and then cell survival was determined using clonogenic assay. (C) HCT116-Mock and-TPP1 cells were irradiated with 6 Gy X-ray and recovered for indicated times. Cell cycle was analyzed by FACS. (D) The population of cells in G2/M phases over time in HCT116- Mock and -TPP1 cells.

To examine the influence of TPP1 overexpression on cellular radiosensitivity, HCT116-TPP1 and HCT116-Mock cells were established (Figure 2A) and cell survival was measured by a clonogenic assay. HCT116-TPP1 cells showed significantly radioresistance compared with HCT116-Mock cells after IR exposure (Figure 2B). 

### TPP1 Overexpression in HCT116 Cancer Cells Causes Prolonged G2/M Arrest after IR Exposure

As shown in Figure 2C, TPP1 overexpression had no significant effect on cell cycle distributions in the absence of DNA damage. Following radiation exposure, we observed that the G2/M arrest reached to a peak at 18 h after IR exposure in both HCT116-Mock and -TPP1 cells. More importantly, the kinetics of the response of the cell lines was different. In HCT116-Mock cells, the G2/M peak gradually decreased from 18h after ionizing radiation and returned to normal levels at about 42 h. However, the G2/M peak in HCT116-TPP1 cells did not decrease but still maintained at a high level until 30-36 h after IR. These results suggest that TPP1 overexpression in HCT116 cells prolonged G2/M arrest after IR exposure.

### TPP1-induced G2/M Arrest Prolongation is Mediated by ATM/ATR-Chk1 Pathway

**Figure 3:**
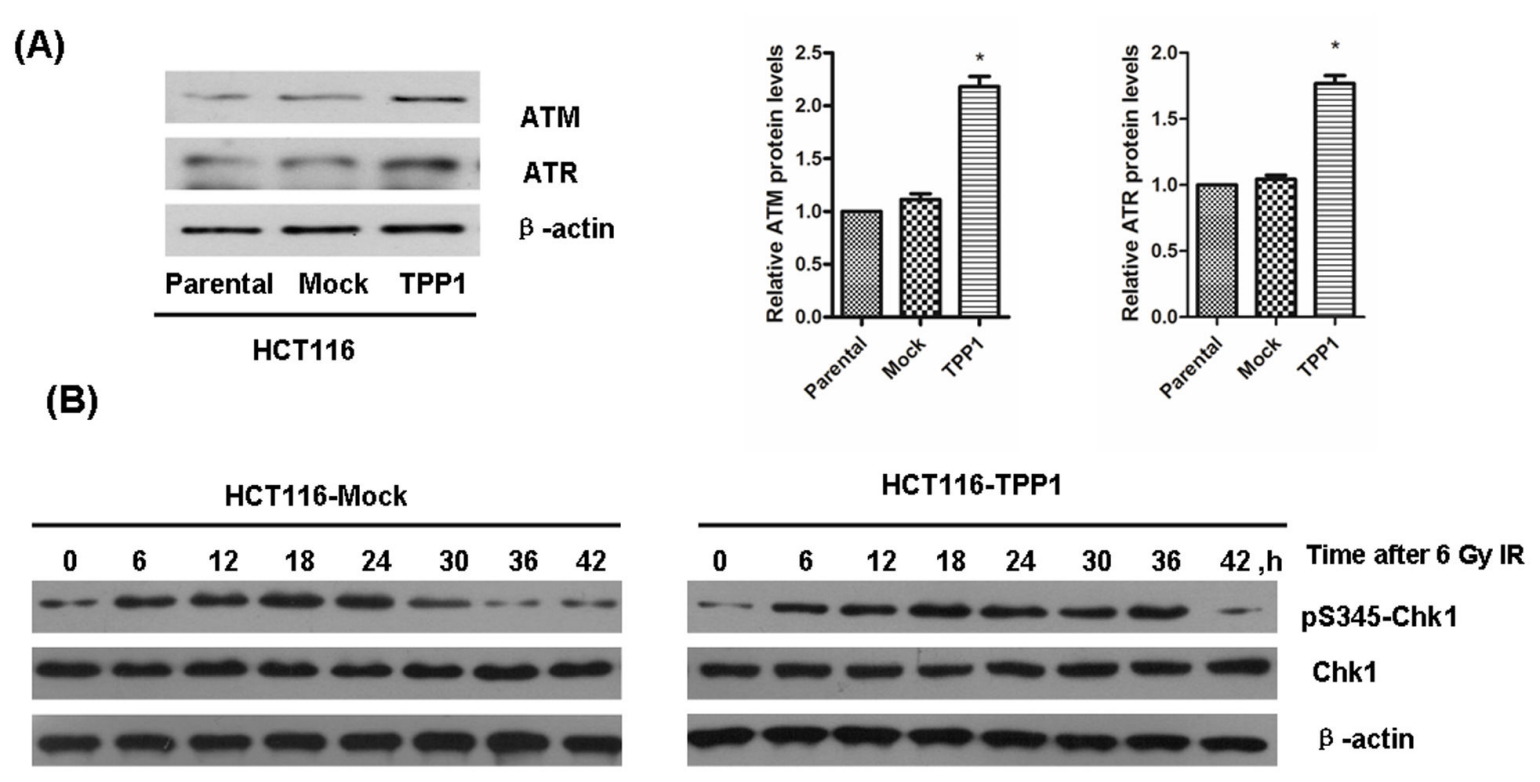
TPP1 overexpression increased **ATM**/**ATR** expression and induced prolonged Chk1 (p^345^) phosphorylation. (A) Western blot analysis revealed that TPP1 overexpression increased the expression of ATM and ATR. (B) HCT116-Mock and-TPP1 cells were irradiated with 6 Gy X-ray and incubated for indicated times. Western blots were preformed to detect the expression of Chk1 and p-Ser^345^-Chk1.

To identify the molecular mechanisms of prolonged G2/M arrest after IR exposure in TPP1-overexpressing cells, we measured the production of ATM, ATR and Chk1. We found that the expressions of ATM and ATR were both elevated in HCT116-TPP1 cells (Figure 3A). Then, we investigated the activations of Chk1, an important substrate of ATR and ATM. We found that phosphorylation levels of Chk1 at Ser345 were higher until 36 h after IR exposure in HCT116-TPP1 cells. In contrast, the levels in HCT116-Mock cells had returned to normal levels at about 30h after IR exposure (Figure 3B). These results indicate that prolonged G2/M arrest by TPP1 overexpression is likely due to ATM/ATR-Chk1 signaling pathway.

### TPP1 Overexpression Inhibits Apoptosis Induced by Ionizing Radiation

**Figure 4:**
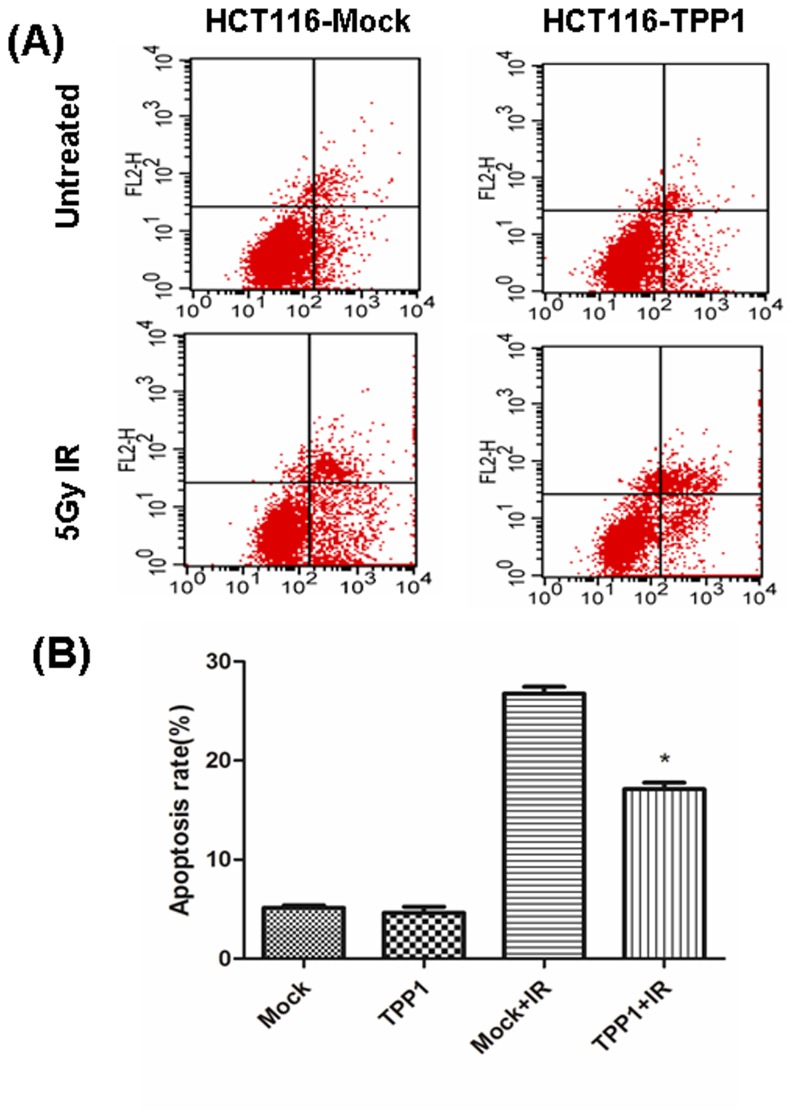
TPP1 overexpression inhibits IR induced apoptosis. HCT116-Mock and-TPP1 cells were irradiated with 5 Gy X-ray and incubated for 24h. The percentage of apoptotic cells was measured by flow cytometry. (A) Representative results of diffrerent groups are shown. (B) Data shown are means±SEM from three independent experiments. *, P < 0.05.

We evaluated the effects of TPP1 on radiation induced apoptosis using flow cytometric analysis. As shown in Figure 4, we found that there was no significant difference between HCT116-Mock and HCT116-TPP1 cells (5.72±0.15% to 5.55±0.12%, p>0.05), but TPP1 overexpression could attenuate the radiation (5Gy) induced apoptosis levels, from 26.89%±0.75 in the control cells to 17.47±0.45% in the HCT116-TPP1 cells (p<0.05). These data indicated that the increased radioresistance by TPP1 overexpression may be due to a decrease in the radiation induced apoptosis.

### TPP1 Overexpression Increases Telomere Length in HCT116 Cells

**Figure 5:**
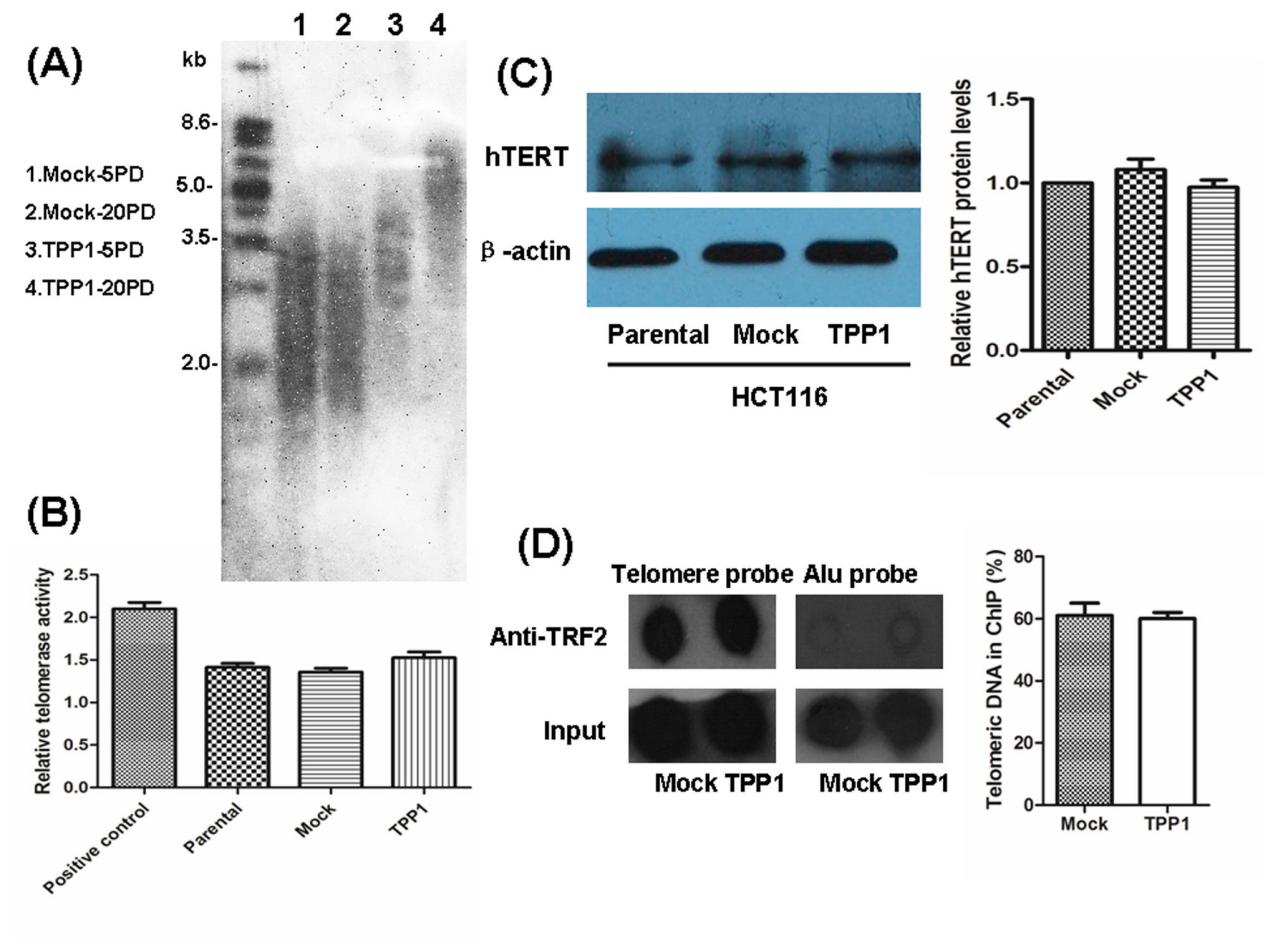
Effects of TPP1 overexpression on localization of TRF2 with telomeres, telomere length and telomerase activity. (A) Mean TRF lengths at different PDs were detected by southern blot. PD, population doubling. The position of MWs (kb) is indicated to the left. (B) TRAP PCR ELISA assay was used in the analysis of telomerase activity at different PDs. (C) Western blot analysis revealed that TPP1 overexpression had no significant influence on the expression of hTERT. (D) Telomere-ChIP assays were performed using a TRF2 antibody to examine the telomeric DNA bound to by TRF2. Input, supernatant before immunoprecipitation; ppt, protein-DNA immunoprecipitate complex. Specific (telomeric) and nonspecific (Alu) probes were used. Telomeric DNA in ChIP (%) =Telomeric DNA signals of ppt / Telomeric DNA signals of input* 100%.

To further investigate the role of TPP1 in telomere length control, HCT116-TPP1 and -Mock cells were cultured for 20 PDs and telomere length was measured by southern blotting. We displayed that the average telomere length of HCT116-TPP1 cells were gradually lengthened than that in the control cells (Figure 5A). These results suggest that TPP1 overexpression could increase telomere length in HCT116 cells.

### TPP1 Overexpression does not Impact Telomerase Activity

To investigate whether the elongated telomeres in HCT116-TPP1 cells were a result of increased telomerase activity, telomerase activity and hTERT protein levels in HCT116-TPP1 cells were compared with HCT116-Mock cells. There was no detectable increase in hTERT protein levels or in telomerase activity in HCT116-TPP1 cells compared with mock cells or parental cells (Figure 5B and C). This result indicates that telomere elongation by TPP1 is not due to an overall increase in telomerase activity. 

### TPP1 Overexpression Accelerated the Repair Kinetics of DNA Damage Induced by IR

**Figure 6:**
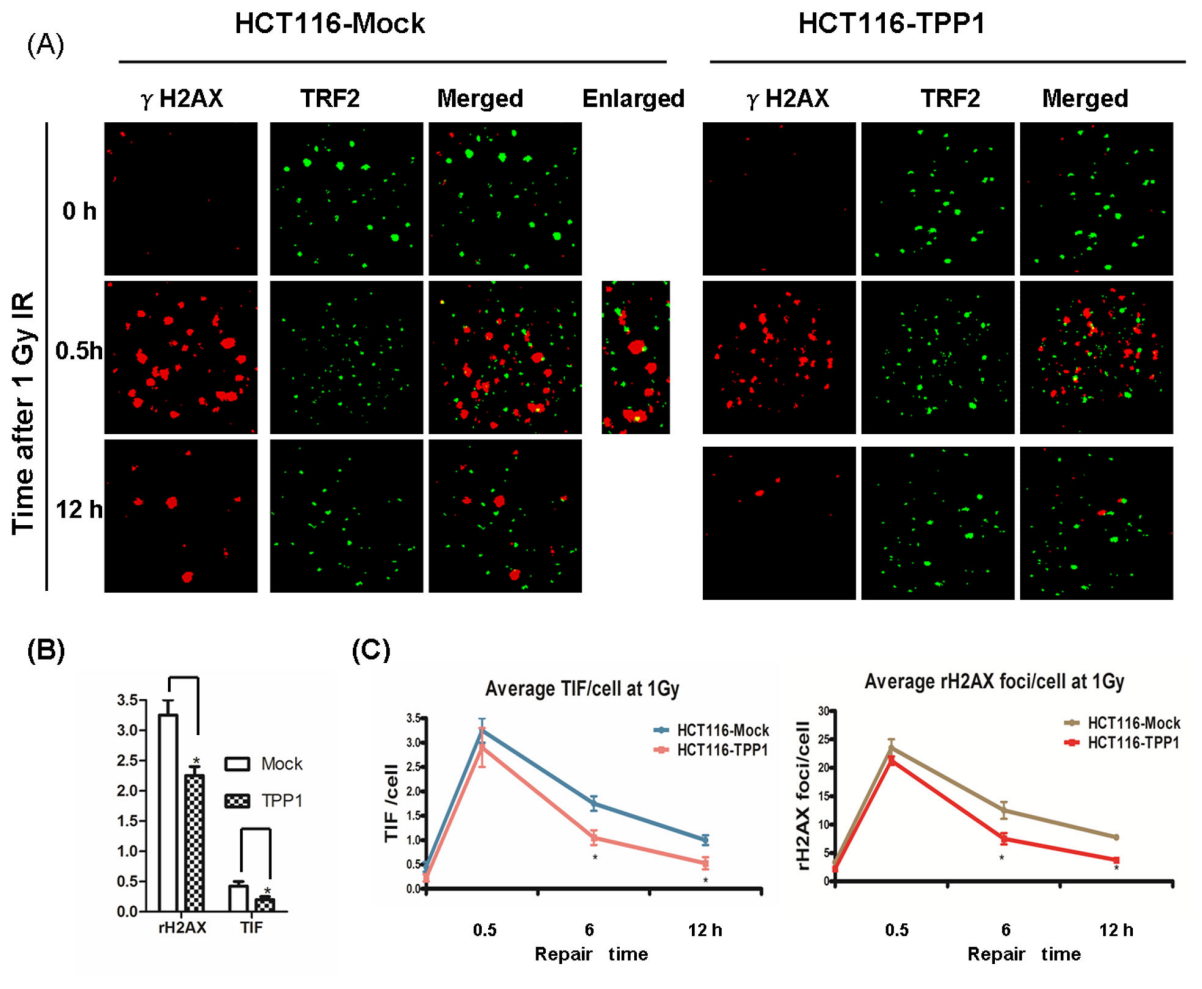
TPP1 overexpression promotes repair of DNA damage and telomere dysfunction induced by irradiation. HCT116-Mock and -TPP1 were exposed to 1 Gy IR and incubated at indicated time points.. Results are based on three independent experiments with on average 100 cell nuclei analyzed per experiment per point. Bars represent the mean± SEM of 3 independent experiments. (A) Representative images for TIFs are shown. (B) Frequencies of spontaneous γ-H2AX positive foci and TIFs in HCT116-Mock and -TPP1 cells. (C) Repair kinetics of IR induced TIF in HCT116-Mock and -TPP1 colorectal cells. Average TIFs per cell at different time points after IR exposure were quantified. (D) Repair kinetics of IR induced DNA damage in HCT116-Mock and -TPP1 colorectal cells. Averageγ-H2AX positive foci per cell at different time points after IR exposure were quantified.

We used TIF assay to establish whether TPP1 overexpression impact repair kinetics of DNA damage at telomeres. Telomere-ChIP assay revealed that TPP1 overexpression had no impact on the association between TRF2 and telomeres (Figure 5D), so TIFs were monitored by co-localization of TRF2 and γ-H2AX in this study (Figure 6A). We observed significantly lower frequencies of spontaneous TIFs in the HCT116-TPP1 cells compared to the control cells (p < 0.05) (Figure 6B).Then HCT116-TPP1 and -Mock cells were exposed to 1 Gy IR and stained to identify the TIF foci at 0.5, 6 and 12 h after IR exposure. Our research implied that TPP1 overexpression cells were able to repair TIFs more efficiently than the control cells. For example, frequencies of IR induced TIFs were similar in HCT116-TPP1 and HCT116-Mock cells 0.5 h after IR, indicating that TPP1 did not reduce the number of TIFs induced by IR. Then TPP1 overexpression cells had approximately 0.53 TIFs/cell 12 h after IR, whereas the mock cells had 1.04 TIFs/cell 12 h after IR (Figure 6C). Thus, HCT116-TPP1 cells showed increased capacity to repair damage at telomeres. The TIF assay identified γ-H2AX foci at telomeres, as well as total γ-H2AX foci in the nucleus. Then we quantitated the formation of total γ-H2AX foci, a marker for DSBs, to further investigate the underlying mechanism for radioresistance. Similar with the results of TIFs assay, the rate of total DNA double-strand break repair was accelerated by TPP1 overexpression. These data indicates that TPP1 overexpression could accelerate the rate of DNA repair after radiation exposure.

## Discussion

We have demonstrated for the first time, to our knowledge, that TPP1 overexpression is associated with radioresistance in colorectal cancer cells in this work.

TPP1 plays crucial roles in telomere length regulation and DNA damage response. In this study, we demonstrated that TPP1 expression was closely correlated with telomere length and radiosensitivity in colorectal cancer cells. Furthermore, we found that ectopic overexpression of TPP1 led to radioresistance and telomere lengthening in HCT116 cells. Previous researches verified that there was a significant negative correlation between telomere length and radiosensitivity [10,11,13]. Our study indicated that TPP1 involved in radioresistance through the regulation of telomere length. Telomeres play a key role in the maintenance of chromosome integrity and stability, and shortened telomere length is associated with increased risk of cancer [23]. POT1 protein levels are associated with telomere length in gastric cancer [24,25]. TPP1 heterodimerizes with POT1 but there are no results about the correlation between TPP1 levels and telomere length in cancer samples. We think the correlation between TPP1 levels and telomere length in colorectal cancer samples is of great importance. Actually, in our study, we tried to do this work using paraffin specimens but found that fresh cancer tissues are more suitable for the southern blotting experiment. In our following study, we will collect more fresh cancer tissue samples and verify the relationship between TPP1 and telomere length in colorectal cancer using fresh cancer tissues.

We found that TPP1 overexpression prolonged radiation-induced G2/M arrest after IR exposure. Cells arrested in G2/M phase allow more time to repair damage thus confer radioresistance. Activation of checkpoints regulates the arrest of the cell cycle in response to DNA damage. Ataxia telangiectasia (AT) mutated (ATM) and ATM and rad3-related (ATR) protein kinases are major upstream checkpoint kinases for DNA damage response [26]. We revealed that TPP1 overexpression elevated the expressions of both ATM and ATR protein. Previous study showed that increased ATM protein levels correlated with intrinsic radioresistance in GBM tumors [27]. Kim and colleagues found that ATR overexpression led to prolonged G2/M arrest and radioresistance in HCT116 cells [28]. Many studies also demonstrated that inhibition the activity of ATM or ATR could result in increased radiosensitivity [29,30]. Chk1 is an important substrate of ATM and ATR. Moreover, Chk1 is an effective target for radiosensitization in human cancer cells [31,32]. Phosphorylation of Chk1 on S345 is regarded as an indicator of Chk1 activation. In this paper, we found that Chk1 phosphorylation was elevated and sustained until later time points after IR exposure in TPP1-overexpressing cells compared with the mock cells. Our study may indicate that prolonged G2 arrest by TPP1 is likely due to higher levels of ATM/ATR-Chk1 signal pathway.

Many studies have shown that telomere homeostasis serves as a potential target in cancer treatment, especially in radiotherapy. Telomere homeostasis can be maintained by telomerase as well as their associated proteins (termed as shelterin). Telomere length, telomerase activity and telomere dysfunction are the major markers of telomere homeostasis. Firstly, telomere length analysis showed significant telomere elongation in HCT116-TPP1 cells compared with control cells, indicating that TPP1 may act as a positive regulator of telomere length. However, it was observed that expression of TPP1 had no effect on telomere length in human fibrosarcoma HTC75 cells [16]. The difference between these results may be due to the distinct chosen in cell lines. Interestingly, there was no detectable increase in telomerase activity or hTERT protein levels in HCT116-TPP1 cells compared with control cells. This result indicates that telomere elongation by TPP1 is not due to an overall change in telomerase activity, but may be due to the hTERT nuclear translocation or localized activation of telomerase at the telomere.

Co-localization of telomeres and activated DDR markers(such as 53BP1 andγ-H2AX), so called telomere dysfunction-induced foci (TIF), is a typical mark of telomere dysfunction. TIFs imply DDR of uncapped telomeres [33].Recent studies demonstrated that TPP1 involves in DNA damage response and suppression of TPP1 expression in mouse embryo fibroblasts (MEFs) or human cancer cells could initiate telomere dysfunction [19,20]. In this study, we found that TPP1 overexpression inhibited the spontaneous TIFs in HCT116 colorectal cells. So TPP1 may protect telomere structure and maintain normal telomere function. 

We found that TPP1 overexpression accelerated the repair kinetics of total DNA double strand break induced by IR exposure. More importantly, TIF assay revealed that the repair rate of DNA damage at telomeres following radiation was also accelerated by TPP1 overexpression. Telomere homeostasis had been identified to serve as a potential target in radiotherapy. Studies had revealed that telomerase inhibition could result in telomere dysfunction and thus increased radiosensitivity [22,34]. It was also confirmed that disruption of shelterin could result in telomere dysfunction [14,20]. David Soler and colleagues showed that dysfunctional telomeres in human epithelial cells were likely to interfere with the efficient repair of radiation-induced DSBs and then led to increased radiosensitivity [35]. Our study demonstrated that TPP1 may participate in telomere homeostasis and could protect telomere from radiation in human colorectal cancer cells.

In conclusion, this study reveals that elevated TPP1 levels protect telomere from DNA damage and confer radioresistance in human colorectal cancer cells. In addition, we provide evidence of the correlation between TPP1expression, telomere length and intrinsic radiosensitivity. In addition, this study has advanced the understanding of the relation between telomere homeostasis and radiosensitization. These findings suggested that TPP1 levels may be a useful indicator of responsiveness to radiation therapy. In summary, our study for the first time indicates that TPP1 may be a potential target in the radiotherapy of colorectal cancer. Moreover, TPP1 inhibition TPP1 inhibition may provide a functional adjuvant in radiation therapy, a possibility we are currently investigating.
